# Multi-spectral diffusion MRI mega-analysis in genetic generalized epilepsy: Relation to outcomes

**DOI:** 10.1016/j.nicl.2023.103474

**Published:** 2023-07-08

**Authors:** Barbara A.K. Kreilkamp, Christina Stier, Erik H. Rauf, Pascal Martin, Silke Ethofer, Holger Lerche, Raviteja Kotikalapudi, Justus Marquetand, Peter Dechent, Niels K. Focke

**Affiliations:** a Clinic for Neurology, University Medical Center Göttingen, Göttingen, Germany; b Department of Neurology and Epileptology, Hertie Institute of Clinical Brain Research, University of Tübingen, Tübingen, Germany; c Department of Neurosurgery, University of Tübingen, Tübingen, Germany; d Laboratory for Predictive Neuroimaging, University of Duisburg-Essen, Essen, Germany; e Department of Neural Dynamics and Magnetoencephalography, Hertie-Institute for Clinical Brain Research, University of Tübingen, Tübingen, Germany; f MEG-Center, University of Tübingen, Tübingen, Germany; g Department of Cognitive Neurology, University Medical Center Göttingen, Göttingen, Germany

**Keywords:** TBSS, ComBat, Multi-site, Multi-cohort

## Abstract

**Background and objectives:**

Genetic generalized epilepsy (GGE) is the most common form of generalized epilepsy. Although individual patients with GGE typically present without structural alterations, group differences have been demonstrated in GGE and some GGE subtypes like juvenile myoclonic epilepsy (GGE-JME). Previous studies usually involved only small cohorts from single centers and therefore could not assess imaging markers of multiple GGE subtypes.

**Methods:**

We performed a diffusion MRI mega-analysis in 192 participants consisting of 126 controls and 66 patients with GGE from four different cohorts and two different epilepsy centers. We applied whole-brain multi-site harmonization and analyzed fractional anisotropy (FA), as well as mean, radial and axial diffusivity (MD/RD/AD) to assess differences between controls, patients with GGE and the common GGE subtypes, i.e. GGE with generalized tonic-clonic seizures only (GGE-GTCS), GGE-JME and absence epilepsy (GGE-AE). We also analyzed relationships with patients' response to anti-seizure-medication (ASM).

**Results:**

Relative to controls, we identified decreased anisotropy and increased RD in patients with GGE. We found no significant effects of disease duration, age of onset or seizure frequency on diffusion metrics. Patients with JME had increased MD and RD when compared to controls, while patients with GGE-GTCS showed decreased MD/AD when compared to controls. Compared to patients with GGE-AE, patients with GGE-GTCS had lower AD/MD. Compared to patients with GGE-GTCS, patients with GGE-JME had higher MD/RD and AD. Moreover, we found lower FA in patients with refractory when compared to patients with non-refractory GGE in the right cortico-spinal tract, but no significant differences in patients with active versus controlled epilepsy.

**Discussion:**

We provide evidence that clinically defined GGE as a whole and GGE-subtypes harbor marked microstructural differences detectable with diffusion MRI. Moreover, we found an association between microstructural changes and treatment resistance. Our findings have important implications for future full-resolution multi-site studies when assessing GGE, its subtypes and ASM refractoriness.

## Highlights

• We performed a mega-analysis of whole-brain multi-site diffusion MRI data.
• Diffusion MRI can identify genetic generalized epilepsy (GGE) and GGE-subtypes.
• We did not find effects of duration, frequency or age of onset.
• Patients with refractory GGE had lower anisotropy compared to patients with non-refractory GGE.

## 1 Introduction

Genetic generalized epilepsy (GGE) is a common form of epilepsy, constituting nearly a fifth of all epileptic disorders (Jallon and Latour, 2005). GGE is characterized by seizures that are not attributable to a single focus in the brain but are generated in bilateral networks. Although individual patients with GGE present without discernible brain lesions on MRI, group comparisons using voxel-based morphometric analyses have frequently identified cortical and subcortical atrophy in patients with GGE (Kazis et al., 2021). Bin et al. (Bin et al., 2017) have reported decreased gray matter volumes for GGE in bilateral pulvinar; for juvenile myoclonic epilepsy (GGE-JME) in the right pulvinar and for absence epilepsy (GGE-AE) in the right medial dorsal nucleus, right subcallosal gyrus, left caudate and left precuneus. When comparing patients with GGE-AE and GGE-JME no significant gray matter volume differences were found (Bin et al., 2017).

Another analysis technique is diffusion magnetic resonance imaging (dMRI), which allows quantification of the diffusion characteristics along white matter tracts and is frequently implemented as diffusion tensor imaging (DTI). DTI-derived metrics include fractional anisotropy (FA), that may be interpreted as a measure of axonal density and myelin. Also included are mean diffusivity (MD, reflecting extra-cellular and -axonal space), radial diffusivity (RD, interpreted as measure of myelin) and axial diffusivity (AD, may quantify axonal membrane circumference) as demonstrated previously (Concha et al., 2010, Gross, 2011).

Many single-site studies to date have found significantly altered diffusion metrics between patients with mixed-syndrome GGE and controls (Focke et al., 2014, Liu et al., 2011, Lobato et al., 2018, Knake et al., 2017). However, these results are, partially, divergent (Sinha et al., 2019). It is likely that discrepancies stem from different diffusion imaging protocols across studies, heterogeneity of the studied cohorts and small sample sizes (Sinha et al., 2019, Hatton et al., 2020). In particular, small studies may not be able to employ conservative statistical corrections that include correction over (multiple) contrasts and, thus, may be prone to false positive findings (Alberton et al., 2020). On the other hand, larger multi-cohort studies done in meta-analytic manner (Hatton et al., 2020) often have limited spatial resolution and limitations of the available clinical information, making it difficult or impossible to analyze finer grained clinical strata like GGE-subtypes, seizure freedom / frequency or ASM response. One meta-analysis has reported significantly decreased FA in patients with generalized epilepsy with no alterations identified in MD (Slinger et al., 2016). Recently, FA results for patients with generalized epilepsy were corroborated in a large multi-site study done in the ENIGMA Epilepsy consortium also reporting increased radial diffusivity (RD) in patients (Hatton et al., 2020). A recent study in 34 patients with mixed-syndrome GGE has identified FA to be an imaging marker of pharmacoresistance (McKavanagh et al., 2021). However, confirmation from larger scale multi-site studies using whole-brain and full resolution analyses with more phenotypical depth is still lacking. We have therefore undertaken a comprehensive assessment of dMRI in GGE, its subtypes and relation to pharmacoresistance, i.e. failure of at least two adequate ASM and no seizure freedom at the time of measurement.

To this end, we have analyzed full-resolution multi-site data from controls and patients with unclassified GGE, patients with GGE-AE, GGE-JME and GGE with generalized tonic-clonic seizures alone (GGE-GTCS). We hypothesize that patients with these GGE syndromes will have white matter alterations specific to these subtypes and relate to clinical factors, in particular epilepsy duration, response to anti-seizure medication (ASM) and presence of GTCS.

## 2 Materials and methods

### 2.1 Participants

We collated clinical and imaging data from four cohorts including GGE patients and healthy controls recruited in two German epilepsy centers (Tübingen, Göttingen) between 2008 and 2021. We complied with all relevant ethical regulations and informed consent was obtained from all subjects. The local ethics committees approved of each study. DTI findings from one cohort have already been disseminated (Focke et al., 2014). Moreover, MEG/EEG and cortical thickness findings have also been published for another cohort (Stier et al., 2021, Stier et al., 2022). Diagnosis of GGE was performed by the epileptologists based on the seizure semiology, patient history and EEG findings according to the ILAE epilepsy classification (Scheffer et al., 2017). The official definition of refractory epilepsy is when a patient has failed two or more ASM treatments (Kwan et al., 2010). Here we have analyzed this within a twelve-month period of refractoriness leading up to MRI. In order to also include patients who had failed just a single ASM treatment, we analyzed patients with active epilepsy separately. We excluded all patients and controls that had brain lesions based on neuroradiological assessments. All participants underwent conventional T1-weighted (T1w, MPRAGE) and diffusion MRI (echo-planar imaging) on either 3 T Siemens Trio or Prisma (Erlangen, Germany). All acquisition parameters and a synopsis of image pre-processing procedures are detailed in Table 1.

**Table 1:** Acquisition Parameters and pre-processing in brief for collated studies.

		TG	TT	PT	PG
**General Information**	*MR system*	**T**im Trio	**T**im Trio	**P**risma	**P**risma-fit
	*Site (Germany)*	**G**öttingen	**T**übingen	**T**übingen	**G**öttingen
	*N (C / GGE)*	21 / 26	18 / 6	42 / 21	45 / 13
	*N (GGE-JME/ GGE-GTCS / GGE-AE / Unk)*	14 / 2 / 8 / 2	0 / 3 / 2 / 1	5 / 2 / 10 / 4	2 / 2 / 3 / 6
	*N _(GTCS / noGTCS)_*	6 / 20	5 / 1	18 / 3	10 / 3
	*N _(Controlled GGE / Active GGE)_*	16 / 7	4 / 0	12 / 8	3 / 9
	*N (GGE-nref / GGE-ref)*	16 / 5	4 / 0	12 / 7	3 / 4
	*Head array coil*	8-channel	12-channel	64-channel	64-channel
**T1w**	*Sequence*	3D-MPRAGE	3D-MPRAGE	3D-MPRAGE	3D-MPRAGE
	*TI (ms)*	900	1100	1100	900
	*Flip-angle*	9°	8°	8°	9°
	*TE (ms)*	3.0–3.2	3.03	3.03	3.3
	*TR (ms)*	2250	2300	2300	2250
	*voxel-size (mm^3^)*	1x1x1	1x1x1	1x1x1	1x1x1
	*# volumes*	2	1	1	2
	*Processing*	*co-registration & averaging (FSL)*	–	–	*co-registration & averaging (FSL)*
**dMRI**	*Sequence*	2D-EPI (axial)	2D-EPI (axial)	2D-EPI (axial)	2D-EPI (axial)
	*Flip-angle*	90°	90°	90°	90°
	*TE (ms)*	93	93	93	96
	*TR (ms)*	10 000	9 400	9 400	4 100
	*voxel-size (mm^3^)*	1.9x1.9x1.9	1.8x1.8x1.8	1.8x1.8x1.8	1.7x1.7x1.7
	*b-value (s/mm^2^)*	1000	1200	1200	1000 & 2000
	*# DWI*	30	46 (2x)	46 (2x)	98
	*# non-DWI*	1	6 (2x)	6 (2x)	10 (2x)
	*Pre-processing*	–	topup (FSL)	topup (FSL)	topup (FSL)
		eddy (FSL)	eddy (FSL)	eddy (FSL)	eddy (FSL)
		ANTs	ANTs	ANTs	ANTs

*Note.* TG / TT / PT / PG = site abbreviations as per rows 1&2; C = controls; GGE = genetic generalized epilepsy; GGE-JME = patients with GGE and juvenile myoclonic epilepsy; GGE-GTCS = patients with GGE and generalized tonic-clonic seizures; GGE-AE = patients with GGE and absence epilepsy; GGE-ref = patients with refractory GGE; GGE-nref = patients with non-refractory GGE; ms = milliseconds; mm = millimeters; T1w = T1-weighted; dMRI = diffusion MRI; MPRAGE = magnetization-prepared rapid gradient-echo; TI = inversion time; TE = echo time; TR = repetition time; EPI = echo-planar imaging; 2x = two acquisitions with reversed phase encoding (anterior<>posterior); DWI = diffusion-weighted images; FSL = FMRIB Software Library v6.0.3; ANTs = Advanced Normalization Tools for corrected b0 to T1w anatomical mapping.

We included a total of 126 controls (age in years, mean ± SD = 32.9 ± 12; 73 female) and 66 patients with GGE (23 GGE-AE, 9 GGE-GTCS, 21 GGE-JME and 13 unclassified GGE; age in years, mean ± SD = 31.2 ± 11.2; 43 female). Thirteen patients had unclassified GGE, because they had either a short duration of GGE or insufficient seizure semiology details to allow a clear distinction of the GGE sub-syndrome. The demographics for the GGE-subtype groups were as follows: patients with GGE-AE (mean ± SD = 28 ± 8.7; 15 female), patients with GGE-GTCS (mean ± SD = 32.2 ± 12.8; 5 female) and patients with GGE-JME (mean ± SD = 34.2 ± 11.3; 8 female). Thirty-five patients had medically controlled GGE (mean ± SD = 32.5 ± 11; 20 female), i.e. were completely seizure-free on ASM, and 24 patients had active GGE (mean ± SD = 29 ± 10.9; 9 female) to treatment(s) with ASM in a twelve month period leading up to MRI. In the cohort, 16 patients had refractory epilepsy, (i.e. failing two or more ASM with seizures in a twelve-month period leading up to MRI, mean ± SD = 30.3 ± 11.4; 6 female) and 35 with (at the time of the measurement) non-refractory GGE (mean ± SD = 32.5 ± 11; 20 female). Eight patients had failed just a single ASM treatment course, three patients did not have data for presence of a seizure within twelve months leading up to MRI and four patients were not taking any medication but also did not have seizures. We analyzed 27 patients without GTCS (noGTCS, mean ± SD = 27.9 ± 8; 14 female) and 39 patients with GTCS (mean ± SD = 33.2 ± 12.3; 19 female).

### 2.2 Preprocessing

The diffusion data was preprocessed using FMRIB Software Library (FSL) version 6.0.3 (https://fsl.fmrib.ox.ac.uk/fsl/fslwiki/FSL (Smith et al., 2006). Specifically, we performed geometric distortion correction (Andersson and Sotiropoulos, 2016) and eddy-current and motion correction using FSL's 'eddy' with b-vector rotations (Leemans and Jones, 2009). The b0 images were brain-extracted and motion-corrected, while distortion-correction was achieved by the use of reversed phase-encoding if available (Table 1). The root-mean-square values representing the mean translational and rotational movement across all intra-cerebral voxels from all diffusion-weighted images relative to the first b0 image were extracted from the FSL eddy output files 'eddy_movement_rms' (Kreilkamp et al., 2021). We computed the mean of these values across all diffusion-weighted images for every participant to assess different motion levels between groups. The T1w data was processed using FSL for re-orientation, field-of-view-cropping and brain extraction. ANTs (https://stnava.github.io/ANTs/, version 2.3.1.dev159-gea5a7) was used for bias-field corrections (N4, bspline_fitting_distance = 300, shrink_factor = 3, n_iterations = [50,50,30,20]). The T1w data was aligned into diffusion space using FSL FLIRT (version 6.0) and subsequently we performed non-linear co-registration of the dMRI data to the brain-extracted diffusion space T1w image via ANTs (Syn, [gradientStep = 0.1, updateFieldVarianceInVoxelSpace = 3.0, totalFieldVarianceInVoxelSpace = 1.0], convergence= [5x1x1,1e-6,3], shrink-factors = 2x2x1, smoothing-sigmas = 3x2x1). We did this to fully remove effects of echo planar imaging (EPI) distortions and achieve anatomical alignment of the dMRI data (Wang et al. 2017). Motion and distortion corrected diffusion MRI data were then reconstructed using FSL's DTIFIT.

Tract-based spatial statics (TBSS (Smith et al., 2006)) is a technique that allows cohort-based processing and analyses of whole-resolution diffusion metric maps in the International Consortium for Brain Mapping (ICBM) standard space. Using the default FSL settings, we created multi-site whole-brain TBSS skeletons based on the ICBM FA template with a mean FA skeleton at a threshold of >0.2 (Smith et al., 2006). For every participant we projected the FA map onto the mean FA skeleton. The resulting skeleton diffusion values were harmonized using ComBat (Fortin et al., 2017) correcting for “batch” effects of the four sites, specifically protecting the effect of age and sex and diagnosis group as biological phenotypes of interest. ComBat-harmonized diffusion metrics (fractional anisotropy, FA, mean diffusivity, MD, radial diffusivity, RD, axial diffusivity, AD) were saved as individual modalities for subsequent statistical testing with a total of 128 501 TBSS skeleton voxels per metric at isotropic resolution (Table 1).

### 2.3 Statistical analysis

Disease duration and age of onset for patients with non-refractory GGE were normally distributed, while all other variables such as age, duration and age of onset for patients with refractory GGE and head motion values were non-normally distributed (Lilliefors, p < 0.05). We therefore performed group difference tests in age, clinical variables (age of onset and duration) and head motion during dMRI using the Kruskal-Wallis ANOVA and Mann-Whitney U-tests. A Chi-Square test of independence was used to assess frequencies of sex, subtypes and refractoriness within groups.

Group comparisons based on dMRI data were performed between controls and patients with GGE and among the patients with different subtypes (GGE-AE, GGE-GTCS, GGE-JME; main analysis) and in our sub-analyses according to refractoriness (non-refractory [GGE-nref] and refractory [GGE-ref]), active and medically controlled epilepsy in separate comprehensive statistical tests. Furthermore, we separately analyzed patients with GTCS (GTCS) and without GTCS (noGTCS). Multiple comparison correction (family-wise-error rate, FWE) was performed within the analysis space over contrasts (group comparisons (Alberton et al., 2020), CFWE). We have used permutation analysis of linear models (PALM (Winkler et al., 2014) accounting for age and sex as regressors of no interest. We have also performed PALM to separately test for effect of clinical variables (duration, age of onset and seizure frequency) on anisotropy and diffusivity values in TBSS skeletons again while accounting for age and sex. We ran 500 permutations with tail-approximation to allow for an efficient inference (Winkler et al., 2016). Exchangeability blocks were defined by MRI scanner. Cohen's D effect sizes were also saved.

All results were considered significant at *p*_(CFWE)_ < 0.05 corrected across all tested contrasts. Anatomical locations were determined using the JHU White-Matter Tractography Atlas, the JHU ICBM-DTI-81 White-Matter Labels and, when both atlases provided no anatomical label, anatomical locations were appraised visually. For comparisons among patients with GGE-ref and GGE-nref, we also present some exploratory analysis with spatially FWE-corrected results without additional across-contrasts correction (*p*_(FWE)_ < 0.05).

## 3 Results

### 3.1 Demographics and clinical information

There were no significant differences in age (U (N_C_ = 126, N_GGE_ = 66) = 3636.5, *p* = 0.2) or sex (*X*^2^_(1,192)_ = 0.94; *p* = 0.3) between patients with GGE and controls. Kruskal-Wallis ANOVA among controls and patients with all GGE subtypes did not reveal any significant differences for age (*X*^2^_(3,179)_ = 3.4, *p* = 0.33) and sex was equally distributed (*X*^2^_Yates(3,179)_ = 1.5 *p*_Yates_ = 0.47). There were no significant differences in age when comparing patients with controlled GGE and those with active GGE (U (N_Active GGE_ = 24, N_Controlled GGE_ = 35) = 395, *p* = 0.13). Sex distribution showed a trend-level difference between active and controlled GGE (*X*^2^_(1,59)_ = 2.9, *p* = 0.09). Hence, we included sex as regressor of no interest in all our analyses. We found a trend-level difference in age when comparing patients with GTCS and those without (U(N_GTCS_ = 39, N_noGTCS_ = 27) = 410, *p* = 0.06). As a consequence, we also included age as a regressor of no interest in all our analyses. Among patients with GTCS and those without, male and female participants were equally distributed (*X*^2^_(1,66)_ = 0.06, *p* = 0.8). We found no significant difference in age when comparing patients with refractory GGE to those with non-refractory GGE (U(N_GGE-ref_ = 16, N_GGE-nref_ = 35) = 241, *p* = 0.22). Sex was also not significantly different between patients with refractory and non-refractory GGE (*X*^2^_(1,51)_ = 1.7, *p* = 0.2).

Patients with active and controlled GGE did not differ significantly in age of onset (U (N_Active GGE_ = 24, N_Controlled GGE_ = 35) = 436.5, *p* = 0.3) and duration (U (N_Active GGE_ = 24, N_Controlled GGE_ = 35) = 446.5, *p* = 0.35). Patients with GTCS and those without did not differ significantly in age of onset (U(N_GGE-GTCS_ = 39, N_GGE-noGTCS_ = 27) = 213.5, *p* = 0.18) but there was a trend-level effect of duration (U(N_GGE-GTCS_ = 39, N_GGE-noGTCS_ = 27) = 176.5, *p* = 0.08). We therefore repeated our PALM analysis for these groups with duration as a covariate and this did not yield any significant differences between patients with GTCS and those without. Patients with refractory GGE and those with non-refractory GGE did not differ significantly in age of onset (U(N_GGE-ref_ = 16, N_GGE-nref_ = 35) = 111, *p* = 0.14) and duration (U(N_GGE-ref_ = 16, N_GGE-nref_ = 35) = 139.5, *p* = 0.45).

Active/controlled GGE was equally distributed among subtypes including GGE unclassified (*X*^2^_Yates(3,190)_ = 1.8, *p*_Yates_ = 0.6). Refractory/non-refractory GGE was equally distributed among subtypes including GGE unclassified (*X*^2^_Yates(3,51)_ = 2.1, *p*_Yates_ = 0.55). We did not compare the distribution of presence/absence of generalized seizures, because these would be biased by the GGE-GTCS subtype.

### 3.2 Motion assessments

The descriptive statistics for motion values (averaged root-mean-square) computed from the diffusion-weighted images were as follows: controls (mean ± SD = 0.76 ± 0.4), patients (mean ± SD = 0.8 ± 0.4), patients with GGE-AE (mean ± SD = 0.7 ± 0.2), patients with GGE-JME (mean ± SD = 0.8 ± 0.4), patients with GGE-GTCS (mean ± SD = 0.9 ± 0.5). The Kruskal-Wallis ANOVA among controls and all patient subgroups (GGE-AE/GGE-JME/GGE-GTCS) did not reveal any significant differences in these motion values (*X*^2^_(3,177)_ = 0.14, *p* = 1). Patients with active (mean ± SD = 0.84 ± 0.37) and controlled GGE (mean ± SD = 0.79 ± 0.42) did not differ significantly in root-mean-square motion values (U (N_Active GGE_ = 24, N_Controlled GGE_ = 35) = 400, *p* = 0.15). Patients with refractory (mean ± SD = 0.8 ± 0.33) and non-refractory GGE (mean ± SD = 0.8 ± 0.42) also did not differ significantly in root-mean-square motion values (U(N_GGE-ref_ = 16, N_GGE-nref_ = 35) = 241, *p* = 0.22). However, we found a significant difference in root-mean-square motion values (U(N_GTCS_ = 39, N_noGTCS_ = 27) = 291, *p* = 0.001) for patients with GTCS (mean ± SD = 0.88 ± 0.37) compared to patients without GTCS (mean ± SD = 0.67 ± 0.37). The group of patients without GTCS, that was also slightly younger than the other (patients with GTCS, trend-level *p* < 0.1), moved significantly less during scanning. For the subsequent analysis involving these two groups we therefore also entered the root-mean-square motion value as a covariate.

### 3.3 Control/patient comparisons and clinical correlations

**Fig. 1:**
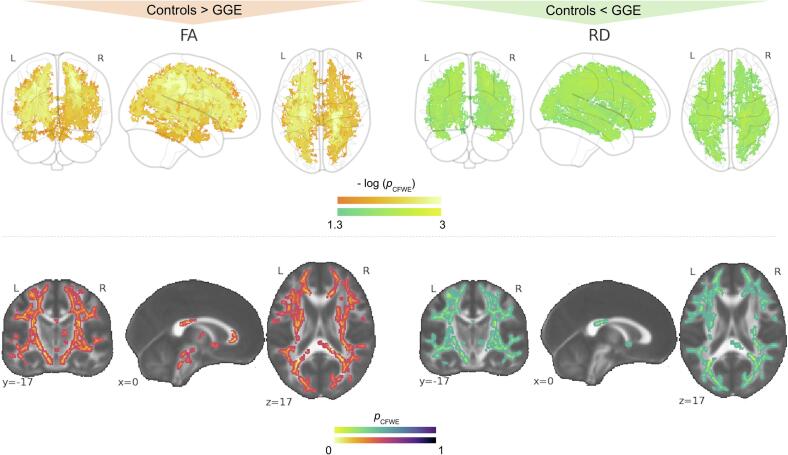
Altered anisotropy and diffusivity metrics in patients with GGE. Results for FA decreases (red-yellow) and RD increases (green-yellow) in patients with GGE compared to controls. All results significant at p_(CFWE)_ < 0.05 and corrected over contrasts. GGE = patients with genetic generalized epilepsy; L = left; R = right; FA = fractional anisotropy; RD = radial diffusivity; CFWE = family-wise-error (contrast corrected). (For interpretation of the references to colour in this figure legend, the reader is referred to the web version of this article.)

Patients with GGE had decreased FA and increased RD values compared to controls and Cohen's D effect sizes reflected this too (Fig. 1, Table 2). At *p_(CFWE)_* < 0.05 the FA and RD alterations merged into large clusters each with 47,435 and 50,284 suprathreshold voxels corresponding to 37% and 39% of the entire TBSS skeleton respectively. Local maxima within the skeletons revealed the most significant reductions (*p_(CFWE)_* = 0.001 with Cohen's D = 0.73 for FA and *p_(CFWE)_* = 0.007 with Cohen's D = 0.35 for RD) in the right cortico-spinal tract. No other significant results were found within FA and RD or within AD and MD maps. There were no significant correlations of seizure frequency, age of onset or duration with any of the diffusion metrics both using the strict *p_(CFWE)_* < 0.05 (corrected over contrasts) or only *p_(FWE)_* < 0.05 correction (corrected just for the analysis).

**Table 2:** Coordinates and anatomical locations of significant alterations across all comparisons with Cohen's D values.

	Metric	# of Voxels	*p_(_*_CFWE)_ < 0.05	X, Y, Z	Cohen's D	Atlas (%)
Controls versus GGE	FA -	47 435	0.001	21, −26, 43	0.73	right CST (38)
	MD	*n.s.*				
	RD +	50 284	0.007	22, −29, 45	0.35	right CST (27)
	AD	*n.s.*				
Controls versus GGE-JME	FA	*n.s.*				
	MD +	10 807	0.022	−34,-71, 20	1.37	left ILF (4), left IFOF (2)
		4 440	0.024	29, −56, 24	1.17	left IFOF (4), left SLF (3), right ILF (2), right CCG (1)
		473	0.048	46, −12, 28	0.96	right SLF (28), right SLFt (7)
		87	0.049	−46, −30, −11	1.07	left ILF (42), left SLFt (8), left SLF (8), left IFOF (8)
		63	0.05	−45, −11, −20	1.11	left ILF (40), left SLFt (4), left SLF (4), left IFOF (2)
		30	0.049	47, −56, 25	1.22	right SLF (4)
	RD +	16 424	0.01	−43, −47, 33	1.4	left SLF (17)
		15 743	0.019	20, −38, 54	1.28	right CST (6)
	AD	*n.s.*				
Controls versus GGE-GTCS	FA	*n.s.*				
	MD -	15 427	0.019	27, −31, 48	1.58	right CST (4) right SLF (3)
		10 972	0.021	−17, −36, 36	1.5	left CCG (2)
	RD	*n.s.*				
	AD -	31 930	0.003	−21, −26, 40	1.5	left CST (37), left SLF (1), left ATR (1)
GGE-AE versus GGE-GTCS	FA	*n.s.*				
	MD -	41 023	0.009	29, −30, 49	1.8	*right PCR*
	RD	*n.s.*				
	AD -	31 690	0.004	19, −37, 29	1.9	right SCC
GGE-GTCS versus GGE-JME	FA	*n.s.*				
	MD +	64 112	0.001	17, −35, 30	2.04	right SCC
	RD +	19 703	0.003	30, 1, 30	2.02	right SLF (6), SLFt (2)
		17 259	0.006	−19, −38, 30	1.97	left CCG (1)
		183	0.048	−34, −3, 22	1.71	left SLF (25), SLFt (11)
	AD +	39 641	0.001	18, −37, 30	0.99	right CCG (1)
		349	0.047	16, 48, 22	1.57	right FM (48), ATR (3), CCG (2)
		123	0.049	−18, −78, 1	1.15	right IFOF (13), ILF (4), FMaj (3)
		33	0.049	29, 31, 32	1.28	*right SCR*
		19	0.05	46, −46, 33	1.33	right SLF (8)
		10	0.05	−17, −39, 57	1.09	left CST (9)
GGE-nref versus GGE-ref	FA -	52	*p_(_*_FWE)_ = 0.046	21,-25,43	1.47	right CST (38)
	MD	*n.s.*				
	RD	*n.s.*				
	AD	*n.s.*				

*Note.* Unique clusters with an extent of at least 10 voxels and with the most significant results at p_CFWE_ < 0.05 are listed. Available anatomical locations were extracted using these atlases: “JHU White-Matter Tractography Atlas”, the “JHU ICBM-DTI-81 White-Matter Labels” (underlined) and through a visual assessment (italics). CFWE = family-wise error correction over contrasts; FA = fractional anisotropy; MD/RD/AD = mean/radial/axial diffusivity; GGE-JME = patients with GGE and juvenile myoclonic epilepsy; GGE-GTCS = patients with GGE and generalized tonic-clonic seizures; GGE-AE = patients with GGE and absence epilepsy; GGE-nref = patients with non-refractory GGE; GGE-ref = patients with refractory GGE; SLF = superior longitudinal fasciculus; SLFt = superior longitudinal fasciculus (temporal segment); CST = corticospinal tract; IFOF = inferior frontal-occipital fasciculus; UF = uncinate fasciculus; ATR = anterior thalamic radiation; SCR = superior corona radiata; PTR = posterior thalamic radiation; ACR = anterior corona radiata; EC = external capsule; CC = corpus callosum; FMaj = forceps major; FMin = forceps minor; CCG = cingulum (cingulate gyrus); PLIC = posterior limb of internal capsule; SCC = splenium of corpus callosum; PCR = posterior corona radiata; CH = cingulum hippocampus. SS = sagittal stratum; ns = not significant.

### 3.4 Control and GGE subtype comparisons

**Fig. 2:**
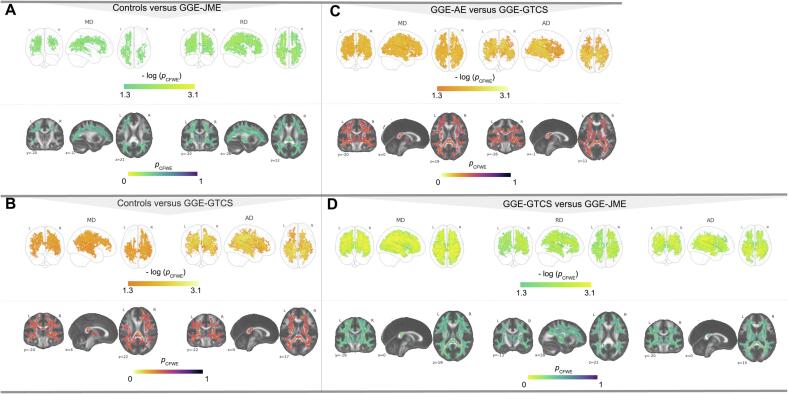
Altered diffusivity metrics in patients with GGE subtypes. Results for increased MD and RD (green-yellow) in patients with GGE-JME compared to controls (A) and results for decreased AD and MD in GGE-GTCS (B). Results for lower AD and MD in patients with GGE-GTCS when compared to GGE-AE (C), increased MD/RD/AD in patients with GGE-JME when compared to GGE-GTCS (D). All results significant at p_(CFWE)_ < 0.05 and corrected over contrasts. GGE = patients with genetic generalized epilepsy; GGE-JME = patients with juvenile myoclonic epilepsy; GGE-GTCS = patients with GGE and generalized tonic-clonic seizures; L = left; R = right; MD/RD/AD = mean / radial / axial diffusivity; CFWE = family-wise-error (contrast corrected). (For interpretation of the references to colour in this figure legend, the reader is referred to the web version of this article.)

In GGE subtype comparisons we found several large clusters of altered diffusion metrics with very large Cohen's D effect sizes such as in the comparison of patients with GGE-GTCS versus GGE-JME (Cohen's D = 2.04, Fig. 2, Table 2). Compared to controls, patients with GGE-JME showed significantly increased MD (10,807 voxels, 8%) and RD (16,424 voxels, 13%). These results were most significant in the left inferior longitudinal and fronto-occipital fasciculi (MD, *p_(CFWE)_* = 0.02) and left superior longitudinal fasciculus (RD, *p_(CFWE)_* = 0.02). Compared to controls, patients with GGE-GTCS had decreased AD (31,930 voxels, 25%) and MD (15,427 voxels, 12%). The most significant results were found in the right corticospinal tract (*p_(CFWE)_* = 0.02, MD), superior longitudinal fasciculus (*p_(CFWE)_* = 0.02, MD) and left corticospinal tract, superior longitudinal fasciculus and anterior thalamic radiations (all *p_(CFWE)_* = 0.003, AD). Patients with GGE-GTCS had lower MD (41,023 voxels, 32%) and AD values (31,930 voxels, 25%) relative to those with GGE-AE. These results were most significant in the posterior corona radiata (MD, *p_(CFWE)_* = 0.009) and right splenium of the corpus callosum (AD, *p_(CFWE)_* = 0.004). Patients with GGE-JME had increased MD/RD/AD compared to those with GGE-GTCS (MD: 64,112 voxels, 49%; RD: 19,703 voxels, 15%; AD: 39 641 voxels, 31%). These values were altered in the splenium of the corpus callosum (*p_(CFWE)_* = 0.001), right superior longitudinal fasciculus (including the temporal segment, *p_(CFWE)_* = 0.003) and right cingulum (*p_(CFWE)_* = 0.001). No other significantly altered clusters were found for MD/RD or AD or within the GGE-subtype FA comparisons.

### 3.5 Other subgroup comparisons

We did not find any significant differences for the investigated metrics when comparing patients with active GGE and those with controlled epilepsy directly both using the strict *p_(CFWE)_* < 0.05 (corrected over contrasts) or only *p_(FWE)_* < 0.05 correction (corrected just for the whole-brain analysis).

Moreover, we did not find any significant differences for the investigated metrics when comparing patients with GTCS and those without directly, both using the strict *p_(CFWE)_* < 0.05 (corrected over contrasts) or only *p_(FWE)_* < 0.05 correction (corrected just for the analysis).

**Fig. 3:**
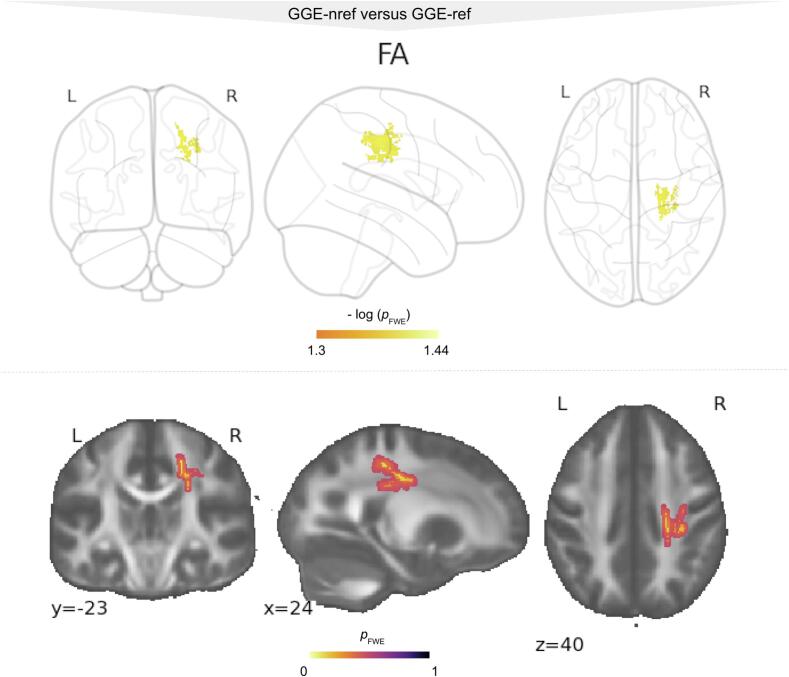
Exploratory analysis without correction over contrasts showing altered anisotropy metrics in patients with non-refractory (GGE-nref) and refractory GGE (GGE-ref). Results for decreased FA (red-yellow) in patients with refractory GGE compared to GGE-nref. All results were significant at p_(FWE)_ < 0.05. GGE = patients with genetic generalized epilepsy; L = left; R = right; FA = fractional anisotropy; CFWE = family-wise-error (contrast corrected). (For interpretation of the references to colour in this figure legend, the reader is referred to the web version of this article.)

Patients with refractory GGE had lower FA values compared to patients with non-refractory GGE with very large Cohen's D effect sizes (Cohen's D = 1.47, Fig. 3, Table 2). The results were not significant when performing spatial- and across-contrast corrections simultaneously. However, at *p_(FWE)_* < 0.05 the FA alterations formed a small cluster of 52 suprathreshold voxels corresponding to 0.04% of the TBSS skeleton. This cluster revealed the most significant reductions (*p_(FWE)_* = 0.046) in the right cortico-spinal tract. No significant results were found for other FA TBSS regions or MD, RD and AD.

## 4 Discussion

We have conducted a multi-site mega-analyses study on whole-brain and full-resolution DTI data. We have demonstrated widespread bilateral anisotropy and diffusion alterations for patients with GGE when compared to controls and among different GGE subtypes. We have not found a significant effect of longer disease duration, higher seizure frequency or younger age of onset on any diffusion metric, which indicates that diffusion metric alterations may not be strongly impacted by the chronicity of the disorder but are related to seizure type instead. Furthermore, we have shown that diffusion values measured in patients with refractory and non-refractory GGE differ from each other.

### 4.1 Biological implications

Compared to controls, patients with GGE as a whole had reduced anisotropy and increased RD. The most significant clusters were found in the right corticospinal tract (Table 2). Overall, our results corroborate previously demonstrated decreased anisotropy in patients with GGE such as white matter within the cingulum and superior longitudinal fasciculus (Sinha et al., 2019). This may indicate reduced myelin in these tracts. Our results are also in line with a previously published whole-brain region-of-interest analysis (Hatton et al., 2020). In keeping with Long et al. (Long et al., 2021) and Sinha et al. (Sinha et al., 2019) we also did not find significant effects of onset age or duration of epilepsy on diffusion metrics. Kim et al. (Kim et al., 2012) could not establish a correlation between duration and diffusion metrics when only analyzing patients with JME. A correlation between cortical alterations and duration and age of onset has also not been established (Woermann et al., 1998). These results may indicate that GGE is not dependent on the chronicity of the disorder.

Moreover, we have shown various white matter alterations between GGE subtypes. Compared to controls, patients with GGE-JME showed the largest cluster of alterations in increased MD within the left inferior longitudinal and frontal-occipital fasciculi and increased RD within the left superior longitudinal fasciculus. This result is in line with previous work demonstrating reduced axial diffusivity within the left superior longitudinal fasciculus (Gong et al., 2017) and reduced FA in bilateral superior/inferior longitudinal and frontal-occipital longitudinal fasciculi (Domin et al., 2018). These results together may indicate increased inter-axonal space due to altered myelination or white matter atrophy (Nagy et al., 2016).

Patients with GGE-GTCS showed decreased MD in the right corticospinal tract and superior longitudinal fasciculus and reduced AD in the left corticospinal tract, superior longitudinal fasciculus and anterior thalamic radiations. The result for AD is corroborated by Liu et al. (Liu et al., 2019) finding of lower FA in the left superior longitudinal fasciculus as measured by diffusion kurtosis imaging. Furthermore, within this group of patients, they report no correlation of duration with any diffusion metric.

Patients with GGE-AE were not significantly different from controls with the stringent error correction levels applied in our study. A study on cerebral volumes only identified abnormalities in five out of twenty patients with absence epilepsy (Woermann et al., 1998). Overall, patients with GGE-AE seem to be underrepresented in the epilepsy imaging literature. Patients with GGE-GTCS had reduced MD and AD when compared to patients with GGE-AE and these changes were seen in the right posterior corona radiata and splenium of the corpus callosum respectively. These alterations may indicate axonal damage in patients with GGE-GTCS.

Patients with GGE-GTCS had increased MD in the right splenium of the corpus callosum, increased RD in the right superior longitudinal fasciculus and increased AD in the right forceps major, anterior thalamic radiations and cingulum when compared to patients with GGE-JME. The most widespread (MD with 64,112 suprathreshold voxels) and most significant (*p_(CFWE)_* = 0.001) alterations were seen in patients with GGE-GTCS compared to GGE-JME. With these findings we provide more evidence for the possibility that disruption of white matter integrity may be the underlying mechanism responsible for myoclonus in GGE-JME (Liu et al., 2011). However, a comparison of callosal morphology based on T1w data did not reveal any differences between GGE-JME and GGE-GTCS (Anastasopoulou et al., 2017).

Previous literature has predominantly identified frontal and thalamo-frontal white matter disruptions in GGE-JME (Kim, 2017, Vulliemoz et al., 2011, Domin et al., 2018). Other studies have also demonstrated more widespread structural alterations in GGE-JME compared to patients with GGE-GTCS (Liu et al., 2011). Patients with GGE-AE and controls did not differ in dMRI-derived values and so patients with GGE-AE show the least white matter disruption. The decrease of FA values found in all patients with GGE may indicate white matter degeneration due to loss of axons, reduced axonal density and altered myelination (Gross, 2011). A decrease in AD (and MD) found in patients with GGE-GTCS when compared to controls or patients with GGE-AE indicates that the magnitude of diffusion parallel to white matter fibers is reduced. This may mean that patients with GGE-GTCS have axonal injury, reduced axonal caliber or a less coherent orientation of axons and it is thought that AD is influenced less by changes in myelin (Gross, 2011, Song et al., 2002).

From a clinical standpoint, our diffusion results may reassure clinicians that GGE subtypes are indeed corresponding to the microstructure of the brain in individual GGE subtypes. However, it remains unclear if the microstructural damage is the cause for a certain type of GGE or whether the damage is secondary to certain types of seizures or their treatment.

Patients with refractory GGE had significantly decreased anisotropy values in the right cortico-spinal tract when compared to patients with non-refractory GGE. This corroborates findings reported earlier in a smaller cohort where patients with refractory GGE had more abnormal network edges (decreased FA) than patients with non-refractory GGE (McKavanagh et al., 2021) when compared to controls. And although alterations between patients with refractory and non-refractory GGE were not significant, this study reported several abnormal network edges with very large Cohen's D values in FA when patients with refractory epilepsy were directly compared to patients with non-refractory epilepsy. These results indicate that patients with refractory GGE have more widespread network disruptions and these may make them less responsive to ASM treatment. However, it remains unclear whether network disruption is the cause or consequence of refractoriness. Kim et al. (Kim et al., 2014) have demonstrated a greater reduction of medial prefrontal functional connectivity in relation to longer disease duration. The authors suggest that thalamoprefrontal network abnormality, the proposed pathophysiologic mechanism underlying GGE, may be the consequence of the long-standing burden of the disease. This could indicate that seizures have caused damage to the brain network and as a consequence patients become refractory to treatment. However, not every study has shown a correlation with duration or seizure frequency. So it may also be the case that patients with a disrupted network are refractory to treatment because of their microstructural alterations. Other previous DTI research on patients with refractory and non-refractory GGE within a mixed GGE cohort of forty patients have yielded no results (Lobato et al., 2018). Further larger studies are needed to identify significant structural alterations with potential as a future imaging marker of ASM-treatment outcomes.

### 4.2 Limitations

The mega-analyses methods used in our study have allowed us to address many contemporary methodological challenges, however, some limitations remain. For instance, although care has been taken to increase sample sizes through collating four epilepsy cohorts, some of the GGE subtype groups remained rather small and this may have impacted on the sensitivity of our statistical analyses. There may have been differences in attending our university center epilepsy clinics in female and male patients, which may have biased study recruitment and sample sizes.

Finally, there is still an open debate on which statistical modeling approach is best when accounting for batch effects (Zugman et al., 2020). We have decided to use ComBat, which was originally developed to account for batch effects in genomics data (Johnson et al., 2007) and has been adapted to provide harmonization for dMRI data (Fortin et al., 2017). ComBat is now commonly used in patient cohorts with epilepsy (Hatton et al., 2020, Sisodiya et al., 2020, Whelan et al., 2018), other neurological disorders (Villalón-Reina et al., 2020, Zavaliangos-Petropulu et al., 2019, Zugman et al., 2020) and its functionality is still being extended (Pomponio et al., 2020).

### 4.3 Conclusions

We were able to identify hints for altered white matter myelination and structure across subtypes. In patients with and without pharmacoresistance we have shown that fractional anisotropy differs significantly. This will present excellent progress when assessing pathobiological alterations across different groups or individuals with GGE and indicates a potential for identifying non-responders of ASM treatments. We demonstrated that a mega-analysis with harmonized diffusion metrics is feasible even in the face of large time gaps between cohorts and differences in acquisition protocols. The results from our mega-analysis are consistent with previous literature conducted in smaller cohorts with isolated GGE subtypes and indicate that patients may be stratified by type of GGE using metrics computed from diffusion imaging. These findings will be relevant for larger multi-site studies aiming to classify patients with GGE, GGE subtypes and according to ASM-refractoriness e.g. using machine learning.

### CRediT authorship contribution statement

**Barbara A.K. Kreilkamp:** Conceptualization, Methodology, Data curation, Software, Writing – original draft, Writing – review & editing, Validation. **Christina Stier:** Methodology, Data curation, Software, Writing – review & editing, Validation. **Erik H. Rauf:** Data curation, Writing – review & editing. **Pascal Martin:** Data curation, Writing – review & editing. **Silke Ethofer:** Data curation, Writing – review & editing. **Holger Lerche:** Data curation, Writing – review & editing. **Raviteja Kotikalapudi:** Data curation, Writing – review & editing. **Justus Marquetand:** Data curation, Writing – review & editing. **Peter Dechent:** Data curation, Writing – review & editing. **Niels K. Focke:** Conceptualization, Methodology, Writing – review & editing, Validation, Supervision.

## Declaration of Competing Interest

The authors declare that they have no known competing financial interests or personal relationships that could have appeared to influence the work reported in this paper.

## Data Availability

Data will be made available on request.
